# Clinical evaluation of the PowerGlide Pro midline catheter– dwell time, complications and outcomes for various medications including prostaglandins

**DOI:** 10.1007/s00423-024-03546-y

**Published:** 2024-11-27

**Authors:** Yaser Souri, Edgar Franklin Hernandez Cancino, Hagen Kerndl, Alexander Hyhlik-Duerr, Yvonne Gosslau

**Affiliations:** Vascular Surgery, Faculty of Medicine, University of Augsburg, Stenglinstr. 2, 86156 Augsburg, Germany

**Keywords:** Midline catheter, I.V. access, Prostaglandins, Dwell time, Peripheral artery disease

## Abstract

**Purpose:**

The PowerGlide Pro™ Midline Catheter is a peripheral venous access device with a length of 8–10 cm, allowing the tip to reach far into the venous system. The aim of this study was to evaluate the dwell time of the catheter. Secondary endpoints included suitability for specific medications (e.g. prostaglandins) and assessment of complications.

**Methods:**

Between January 2019 and November 2021, 50 patients were included in the study. Data on patient demographics, placement characteristics, complications and reasons for removal, were collected.

**Results:**

Placement was technically successful in 92% (*n* = 46) of cases. In all cases, veins of the upper extremity were punctured (34 basilic veins, 7 brachial veins, 6 cephalic veins, and 3 median cubital veins). The average dwell time was 6.1 days (1–17 days). A significant difference between duration and medication administered could not be demonstrated.

**Conclusions:**

The longer maximum dwell time compared to a standard peripheral venous catheter makes it particularly suitable for intravenous therapy for more than 7 days or patients who have poor peripheral vein status.

## Introduction

Establishing venous access through a catheter is one of the most widely used procedures in the hospital setting [1]. Determinants for the type, size, and location of venous access include the clinical status of the patient, the planned therapeutic agents, and the continuous need for laboratory investigations [2]. Moreover, the selection of a venous access device should be based on specific indications for that device, with the goal of minimizing the chances of insertion failure and reducing the possible complications in mind [3].

There are numerous ways of establishing intravenous (IV) access. A peripheral venous catheter (PVC) is inserted in veins located distally on the upper or lower limbs [4]. A central venous catheter (CVC) is a catheter with a tip reaching centrally-located veins in the neck, chest, or groin, including the superior vena cava, inferior vena cava, brachiocephalic veins, internal jugular veins, subclavian veins, iliac veins, and common femoral veins [5]. A peripherally inserted central catheter (PICC) is a form of venous access that extends through a peripheral vein in an extremity to reach a larger central vein [6].

A midline catheter is a peripheral venous access device, usually 8–20 cm in length, which is placed in a vein in the upper arm and extends to or below the level of the axillary vein but does not reach a central vein [1, 7]. The main difference between a midline catheter and a CVC or a PICC line is that the latter two extend to reach central veins, such as the subclavian. Midline catheters are inserted using the Seldinger’s technique, usually with the assistance of ultrasound [8].

Compared to PVC studies have shown that a midline catheter has a lower phlebitis rate [9] and a longer dwell time [2]. Therefore, the insertion of a midline catheter has been recommended when the duration of therapy exceeds six days [10]. When compared to CVC it was found that the use of midline catheters resulted for example in statistically significant lower rates of catheter-related infections (3.5% vs. 0.2%) and was associated with an overall lower mortality rate (5.3% vs. 17.3%) [11].

The typical patient population in vascular surgery is often older and has multiple comorbidities, therefore it is essential to have IV access options that are both comfortable and effective. The tunica intima of veins can be irritated by the application of prostaglandins (Alpostadil, Prostavasin), which is why more central veins are preferred. This is due to the larger diameter of the more centrally located veins, which results in less irritation of the vein wall. As the tip of the Midline catheter is more central than the PVC, we wanted to evaluate the possibility of administering this type of medication via this access. These obstacles are what motivated the current study, which aimed to investigate the use of midline catheters as a viable option for long-term IV access in the Vascular Surgery department at the University Clinic Augsburg.

The main objective of this study is to analyze the dwell time of midline catheters (Power Glide Pro™, BD medical company, Franklin Lakes, United Sates of America) in patients with vascular disease. Secondary endpoints were the feasibility to apply certain medications (e.g. prostaglandins) via midline catheter and the occurrence of site complications.

## Materials and methods

### Study design

This is a single-center prospective observational study that aims to describe the data collected on patients receiving midline catheter insertions. This study was performed in the University Clinic Augsburg (Universitätsklinikum Augsburg) in the time frame between January 1st, 2018, and November 30th, 2020.

As patient consent was required for participation, Clinical Ethics Committee approval was granted after the study protocol was evaluated (Ludwig-Maximilians-Universität Munich project number: 18–813). The study was registered at ClinicalTrials.gov (NCT06037525).

A total of 50 patients were recruited. These Patients had a midline catheter inserted for one of the following reasons: The need for IV access with an expected duration of 7 days or more, the need for Alprostadil (brand name Prostavasin, UCB S.A., Belgium) therapy or Patients with difficult standard PVC insertions due to poor conditions of the veins or without a visible or palpable vein for the insertion of a standard PVC.

The data was collected using three forms filled out by the healthcare provider responsible for the insertion, maintenance, and removal of the midline catheter. Other patient-related data, including clinical notes, laboratory results, and comorbidities, were collected by examining patients’ medical records and stored digitally. Pain during insertion was assessed using the Visual Analogue Scale (VAS).

### Procedure: insertion and maintenance

The procedure of insertion of the Power Glide Pro™ Midline Catheter (Becton Dickinson, Erembodegem, Belgium) was based on the “Instructions for Use.” The conductors of the study were trained by personnel from BD on the proper techniques for the insertion of a PowerGlide Pro™ Midline Catheter. The conductors of the study performed multiple successful midline catheter insertions under supervision before commencing the study. All catheters were inserted by experienced surgeons. Two catheter sizes were used: 18 G and 20 G, along with two lengths: 8 cm and 10 cm. The insertion, maintenance, and removal processes were similar to previous studies [12]. The catheter was mainly ultrasound guided inserted. After insertion the catheter was fixed in place with a special plaster (StatLock Stabilization Device). The StatLock Stabilization Device works by clamping the catheter in place, ensuring that it stays in the desired position. After insertion, a standard catheter care was performed including daily clinical checks for signs of infection or thrombosis, Dressing changes every 7 days or after signs of contamination, and Catheter flushing with 10 ml NaCl after every medication administration to avoid clogging. Furthermore, a weekly laboratory check (leukocyte count and CRP) and ultrasound checks of the catheterized vein for signs of thrombosis or thrombophlebitis were performed.

The price of one catheter depends on the quantity purchased and purchasing conditions may vary locally. During our study, a catheter cost around 20 euros.

### Procedure: removal

The catheter was properly removed after completion of therapy or if complications arise that necessitate the removal of the catheter. Catheter-related pain, extravasation, and occlusion were documented.

The removed catheter and the tip were sent to the microbiology department for examination. A macroscopic assessment of the catheter tip observing for signs of a thrombus formation was carried out directly by the removing health care provider.

### Statistical analysis

All patient data were recorded on a Microsoft Excel sheet. Statistical tests, including regression analyses and Mann-Whitney tests, were carried out on the SPSS software. Findings are presented in the format of median or mean ± standard deviation if appropriate.

## Results

**Fig. 1:**
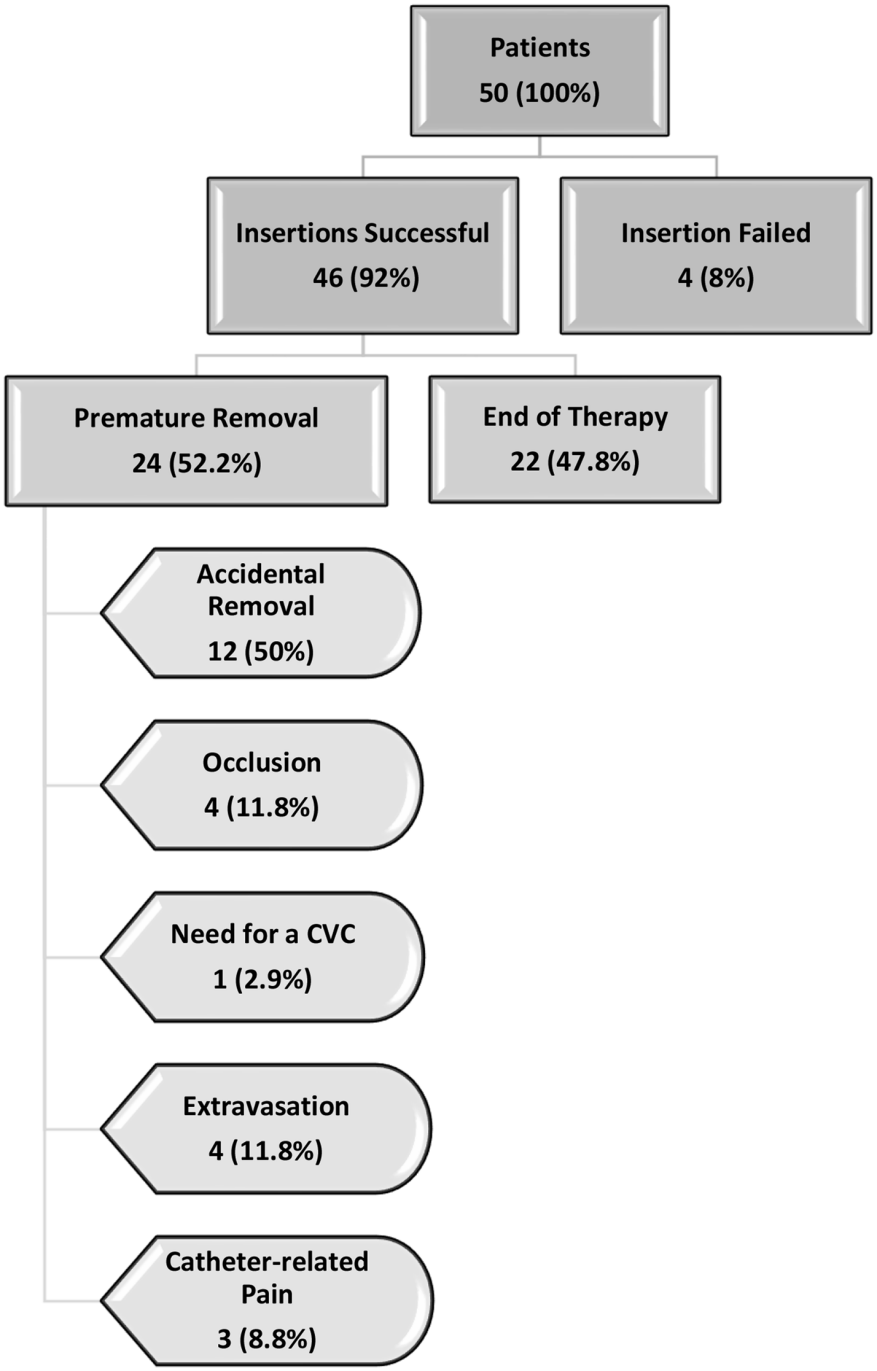
Summary of all patients included in the study (*n* = 50)

Overall, 46 patients were catheterized successfully, meaning the success rate was 92%. The median number of trials required for successful catheterization was one. Figure 1 summarizes all the patients included in the study and their outcomes. Patients who failed the insertion process were excluded from subsequent analysis of the study’s primary endpoint.

During the study, 50 patients receiving midline catheter insertions were enrolled. Patient characteristics are shown in Table 1. These characteristics are representative of the patient population usually admitted to the vascular service at the University Clinic Augsburg.

The average depth of the veins used for catheterization was 7.7 ± 3.3 mm. Ultrasound guidance was used for all catheter insertions except for two (96%), which were superficial veins (1 mm and 2 mm) that could be catheterized directly. The average diameter of the veins with the tourniquet applied was 4.7 ± 1.3 mm. The 8-cm catheter was used in 15 patients, while the 10-cm was used for the remaining 35 patients.

Patients pain upon the insertion of the midline catheter was recorded according to a standard visual analog scale. The median pain score across the patient population was 3.

**Table 1:** Patient characteristics of the study population

Patient characteristics (*n* = 50)	
Age (y)	67.4 ± 12.9
Females (n)	19 (38%)
Comorbidities	
Anticoagulants/antiplatelets	48 (96%)
Hypertension	35 (70%)
Cardiovascular conditions*	27 (54%)
Diabetes Mellitus	16 (32%)
Inflammatory** and neoplasms	11 (22%)

* Hypertension, ischemic heart disease, arrhythmia, history of stroke, hypercholesterolemia, history of deep vein thrombosis

** Vasculitis, lupus

Data are presented as mean ± standard deviation or number (n) (%)

The most frequent reason for catheter removal was the end of therapy (47.8%), while the most frequent cause of premature removal was accidental removal by the patients (26.1%). For different reasons, patients whose catheters were removed accidentally were not reincluded in the study after the removal of the catheter.

The overall complication rate was (11/46 = 23.9%). Out of the 46 successful catheterizations, only one patient (2.2%) had local findings of irritation (swelling) near the catheter site. Daily checks of the catheter site yielded no additional findings for the other 45 patients.

**Fig. 2:**
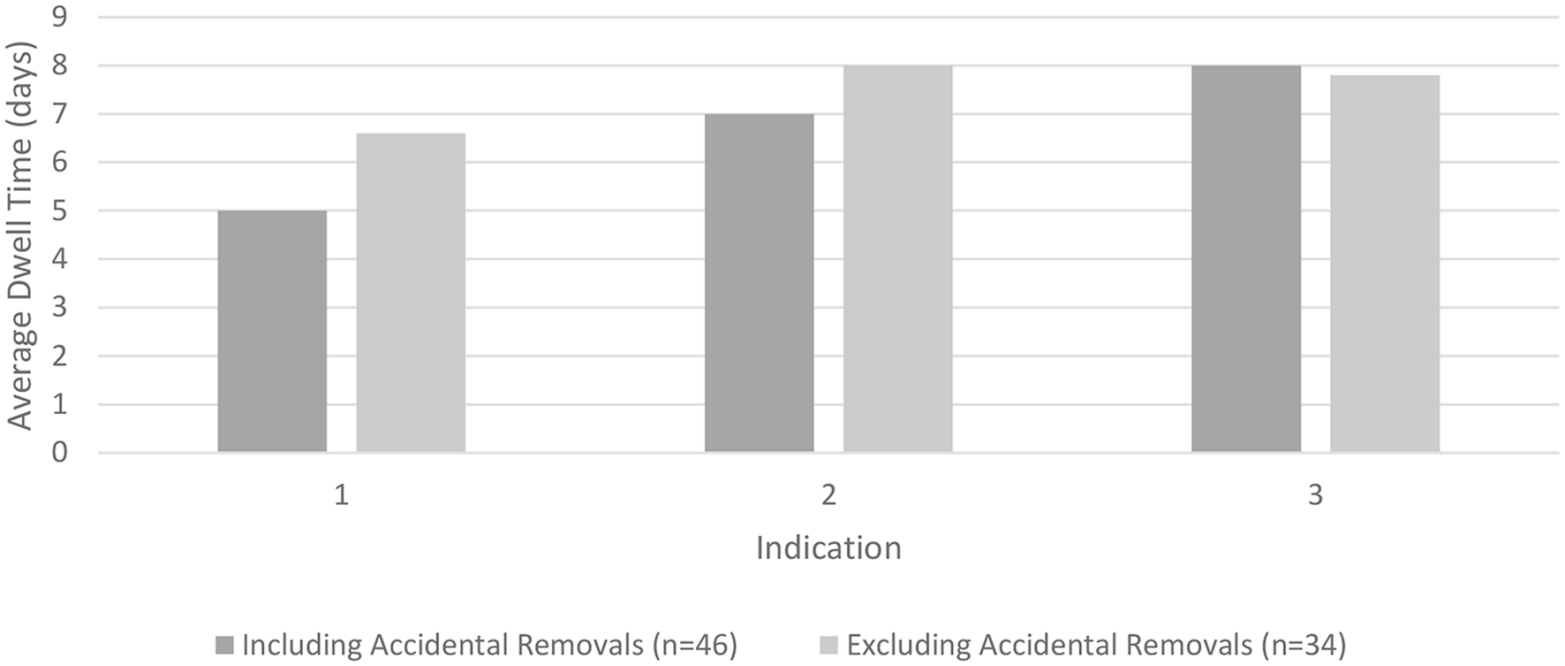
Column chart: Indication for catheter insertion (clustered*) vs. average catheter dwell time (days). 1 = Alprostadil Therapy; 2 = Antibiotic Therapy; 3 = Difficult Standard PVC Insertion. * Clustered means that patients who had more than one indication for insertion were counted as multiple patients for each indication

The average dwell time for the 46 patients with successful catheterizations was 6.1 ± 4.2 days with a median of 5 days (range 1–17 days). When excluding the 12 patients (26.1%) whose catheters were removed accidentally, the average dwell time increases to 7.3 ± 4.1 days, with a median of 6 days. Multiple factors were plotted against the dwell time for the midline catheter. Figure 2 shows that patients who had a difficult standard PVC insertion had the longest average midline catheter dwell times.

Out of 46 catheters, 14 (30.4%) were sent for microbiological examination to check for infection, as they were removed in presence of the investigator. 2 (4.3%) returned positive for contamination. One of the two patients had an inguinal abscess as a complication from known intravenous drug use; the other was a patient with an infected bypass graft. Neither of these patients showed evidence of a catheter-related bloodstream infection. The other 32 catheters were not sent for examination.

**Fig. 3:**
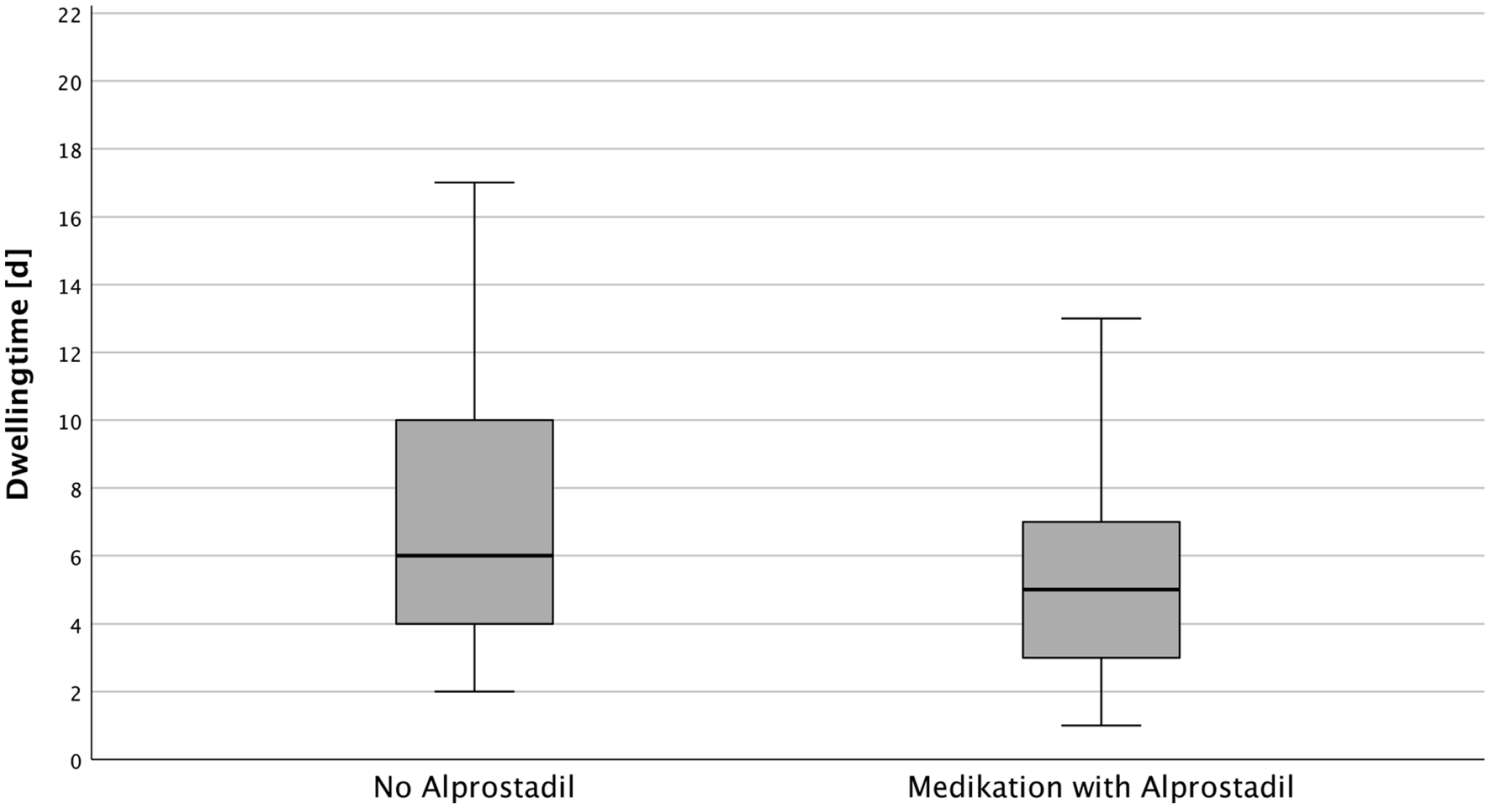
Dwell time (days) by indication: Alprostadil group (*n* = 29): mean dwell time = 5.17 ± 3.56 days. No Alprostadil group (*n* = 17): mean dwell time = 7.76 ± 4.71 days. Mann-Whitney test, *p* = 0.061

33 patients (66%) required a midline catheter for the administration of Alprostadil, 4 of which failed catheterization. The average dwell time for these 29 patients was 5.2 ± 3.6 days, which increased to 6.6 ± 3.3 days when excluding patients whose catheters were removed prematurely by accident. 17 patients did not receive Alprostadil therapy, and their average dwell time was 7.7 ± 4.7 days. The difference in dwell times (2.6 days) showed no significance (*p* = 0.061, Fig. 3).

Out of 29 patients, only one patient had findings suggesting irritation near the injection site (swelling), calculated to be an incidence of 3.4%.

## Discussion

Based on the data presented in the study, it can be concluded that the PowerGlide Pro™ Midline Catheter is a viable alternative to traditional peripheral venous access devices for patients who require intravenous therapy for a longer period or have poor peripheral venous status. The technical success rate of the catheter placement was high (92%), and the procedure was associated with minimal pain. The average catheter dwell time of 6.1 days was longer than that of a traditional peripheral venous catheter, making the PowerGlide Pro™ Midline Catheter a suitable option for patients requiring intravenous therapy for more than seven days.

The catheter is 10 to 15 times more expensive than a standard PVC. Our intention was minimizing this difference in cost by achieving a longer dwell time, where patients normally need more than one venous access device, and thus compensating for the cost difference mentioned above.

The average dwell time reported in our study was comparable to some studies discussing the efficacy of midline catheters [12, 13] but 10 days shorter than the average dwell time of midline catheters in a 2021 study published in the journal of Critical Care Medicine [14]. This variance can be attributed to multiple factors. First, the patient populations receiving the midline catheterizations across the studies were different, with unique demographical characteristics. Moreover, almost half of the patients in our study had their catheters removed due to the end of therapy, suggesting that the catheter was functioning and could have stayed for longer periods of time.

The high volume of accidental removals also shortened the dwell time, as the catheters were functioning before removal. The reason behind the high frequency of accidental removals was the poor patient compliance of the relatively old patient population, and not due to the patients not tolerating this special catheter.

Although no significant difference was found between catheter dwell time and the type of medication administered, the catheter was found to be suitable for the administration of special medications, such as prostaglandins or long-term use of antibiotics.

The infusion of irritant medications peripherally remains controversial due to the lack of robust evidence for the optimal way to administer different medications. Amongst other side effects, Alprostadil is known to cause pain at the injection site [15].

Since the aim of the study was to achieve venous access with a long dwelling time and due to the fact that many patients in the vascular surgery ward receive prostaglandin therapy, we decided to include these patients in the study. Furthermore, since the application of prostaglandins was administered over CVC, which can be associated with serious complications, we tried to establish an alternative for the application of such medications. The availability of an alternative method of administration for Alprostadil that avoids the potential complications of a CVC and the side effects of a PVC can ease the administration of the medication and improve patient outcomes.

Although there were a limited number of patients who received Alprostadil, our study suggests that midline catheters may be a feasible option for administering Alprostadil. Patients who received Alprostadil had shorter average dwell times compared to the rest of the patient population (5.2 days vs. 7.7 days). To evaluate the efficacy and safety of administration of Alprostadil through midline catheters further studies with larger patient populations are needed.

The study also found that the PowerGlide Pro™ Midline Catheter had a lower complication rate compared to central venous catheters, making it a safer alternative for patients.

The midline catheter has many documented complications that may necessitate catheter removal and replacement, including catheter occlusion, infection, venous thrombosis in the ipsilateral arm, extravasation or infiltration of infused liquids, pain, catheter dislodgement, and phlebitis [12, 16]. Our study recorded only occlusion, pain, extravasation, and bloodstream infection.

Overall, our study reported slightly higher rates of occlusion and extravasation but a lower rate of catheter-related pain than other studies. Factors that play a role in this observation may include the relatively older patient population with a high prevalence of comorbidities in our study. In addition, the catheter maintenance protocols, including regular flushing of the catheter every 12 h and after each use, were not strictly adhered to, leading to increased rates of occlusion and catheter dysfunction.

A meta-analysis of 987 articles on the complications of midline catheters found a very low rate of catheter-related infections (0.28/1000 catheter days), with 64% of studies not reporting any infections with the use of midline catheters [14]. This is congruent with our study, where none of the patients had any signs of a catheter-related bloodstream infection. However, the high rate of premature removal and relatively shorter dwell times in our study may underestimate the true incidence of catheter-related infections with the use of midline catheters.

The most common reasons for catheter removal were the completion of therapy and accidental dislodgement. Patients whose catheters were accidentally removed were not demographically different from the rest of the patient population. From a total of 12 patients whose catheters were accidentally removed, 9 received PVCs instead due to the unavailability of personnel who were adequately trained for the insertion of a midline catheter, leading the on-duty physicians to opt for a PVC. One patient declined further treatment and was discharged, and the remaining two patients were disoriented and refused catheterization with the midline catheter.

Overall, the PowerGlide Pro™ Midline Catheter offers an expanded range of options for intravenous therapy, particularly for patients with poor peripheral venous status or who require intravenous therapy for a prolonged period. The catheter can be easily placed using either direct puncture or ultrasound guidance and is associated with minimal pain. With its low complication rate and extended dwell time, the PowerGlide Pro™ Midline Catheter has the potential to reduce the need for central venous catheters in some cases, thereby minimizing the risks associated with more invasive procedures.

### Limitations of the study

Our study was conducted at only one center, whereas a multi-center study might yield more generalizable data. Since we did not compare the midline catheter patient to a control group, this means that our study is purely descriptive, with no ability to draw conclusions on the superiority of different vascular access devices. The presence of only one catheter inserter may have distorted some of the study observations. Moreover, the small number of patients included in the study may affect the validity of conclusions drawn from it. The presence of a large portion of patients whose catheters were accidentally removed also distorts the complete picture of the midline catheter efficiency.

## Conclusion

In conclusion, the findings of this study support the use of the PowerGlide Pro™ Midline Catheter in vascular surgery. The catheter offers a safe and effective alternative to traditional peripheral venous access devices and central venous catheters for patients who require intravenous therapy for a longer period of time or have poor peripheral venous status. Further studies are needed to confirm these findings and to determine the long-term outcomes and cost-effectiveness of using the PowerGlide Pro™ Midline Catheter in clinical practice.

## Data Availability

No datasets were generated or analysed during the current study.
